# Anti-neuron antibody syndrome: clinical features, cytokines/chemokines and predictors

**DOI:** 10.1186/s12974-021-02259-z

**Published:** 2021-12-06

**Authors:** Shaohua Liao, Chuanfen Li, Xiaoying Bi, Hongwei Guo, Ying Qian, Xiaobei Liu, Shuai Miao, Huaiqiang Hu, Bingzhen Cao

**Affiliations:** 1 Department of Neurology, Shanghai Changhai Hospital, Second Military Medical University, Shanghai, China; 2 College of Physical Education, Shandong Normal University, Jinan, China; 3 Department of Neurology, 960 Hospital of the PLA Joint Logistics Support Force, Jinan, China; 4 Department of Neurology, Chinese PLA General Hospital, Beijing, China

**Keywords:** Anti-neuron antibody syndrome, Autoimmune encephalitis, Paraneoplastic neurological syndrome, Cytokines, Chemokines, Predictors

## Abstract

**Background:**

Neuroimmunology is a rapidly expanding field, and there have been recent discoveries of new antibodies and neurological syndromes. Most of the current clinical studies have focused on disorders involving one specific antibody. We have summarized a class of antibodies that target common neuronal epitopes, and we have proposed the term “anti-neuron antibody syndrome” (ANAS). In this study, we aimed to clarify the clinical range and analyse the clinical features, cytokines/chemokines and predictors in ANAS.

**Methods:**

This was a retrospective cohort study investigating patients with neurological manifestations that were positive for anti-neuron antibodies.

**Results:**

A total of 110 patients were identified, of which 43 patients were classified as having autoimmune encephalitis (AE) and the other 67 were classified as having paraneoplastic neurological syndrome (PNS). With regards to anti-neuron antibodies, 42 patients tested positive for anti-N-methyl-D-aspartate receptor (NMDAR) antibody, 19 for anti-Hu, 14 for anti-Yo and 12 for anti-PNMA2 (Ma2). There were significant differences between the ANAS and control groups in serum B cell-activating factor (BAFF) levels and in cerebrospinal fluid (CSF) C-X-C motif chemokine10 (CXCL10), CXCL13, interleukin10 (IL10), BAFF and transforming growth factor β1 (TGFβ1) levels. Predictors of poor outcomes included having tumours (*P* = 0.0193) and having a chronic onset (*P* = 0.0306), and predictors of relapses included having lower levels of CSF BAFF (*P* = 0.0491) and having a larger ratio of serum TGFβ1/serum CXCL13 (*P* = 0.0182).

**Conclusions:**

Most patients with ANAS had a relatively good prognosis. Having tumours and a chronic onset were both associated with poor outcomes. CSF BAFF and the ratio of serum TGFβ1/serum CXCL13 were associated with relapses.

## Introduction

Neuroimmunology is a rapidly expanding field that has led to the discovery of a series of new antibodies and neurological syndromes. Most of the current clinical studies have focused on disorders related to one specific antibody [1–3] and have limitations regarding the understanding of the generalization of these disorders. We have summarized a class of antibodies and have proposed the term “anti-neuron antibody syndrome” (ANAS), which is characterized by antibodies that target common neuronal epitopes in certain clinical neurological syndromes. The target antigens mainly include Hu, Yo, NMDAR, etc. [4–6] The clinical syndromes consist of autoimmune encephalitis (AE) and paraneoplastic neurological syndromes (PNS).

ANAS is considered to be an immune-mediated disorder and is characterised by the presence of antibodies that target neuronal epitopes. T and B cells might be responsible for the syndrome and participate in key steps [4]. Some cytokines/chemokines might play significant roles by affecting the functions of T and B cells. Among anti-inflammatory factors, TGFβ1 and IL10 play important roles by executing their suppressive effects [7, 8]. In proinflammatory elements, CXCL13, CXCL10 and BAFF play crucial roles in the survival, activation and recruitment of T or B cells [9–11]. The accumulated data suggest that the above factors might affect the development of ANAS by interacting with T and B cells.

In this study, we collected the clinical characteristics and data on the cytokines/chemokines in the sera and CSF and aimed to look for the associations between them and ANAS. Then, we strived to identify predictors for the outcomes and relapses of ANAS. Here we report the data.

## Methods

### Study design and participants

We performed a single-centre, retrospective cohort study at the 960 Hospital of the PLA Joint Logistics Support Force. Our study protocol included all patients with neurological manifestations and had positive anti-neuron antibodies, including amphiphysin, CV2, PNMA2, Ri, Yo, Hu, Recoverin, SOX1, Titin, NMDAR and GABA_B_R. Our study included 110 patients hospitalized between March 18, 2011, and July 3, 2017. We collected demographic and clinical data, including age, sex, type of onset, magnetic resonance imaging (MRI) fluid-attenuated inversion recovery (FLAIR)/T2, electroencephalogram (EEG)/electromyogram (EMG), CSF leukocyte counts, CSF protein levels and the antibody-specific diagnosis of the patients. The data with regards to associated tumours, outcomes and relapses were obtained from patients in the hospital and during the follow-up. A total of 108 patients had follow-up evaluations between 24 and 99 months (median 54 months), and 2 patients dropped out. All patients were grouped into AE or PNS by clinical syndromes and into good or poor outcomes by the modified Rankin scale (mRS). They were also grouped into tumours or no tumour and into relapses or no relapse. Good outcomes were defined as a grade of 0–2 on the mRS, while poor outcomes were defined as a 3–6.

The study was approved by the ethics committee of the 960 Hospital (NO.201611). All patients provided informed written consent to allow their medical records and samples of sera and CSF to be used in this study. All patients’ data were processed anonymously.

### Samples, antibodies detection and multiplex cytokines/chemokines immunoassay

A total of 156 samples were obtained from patients within the first 3 days after admission, which included 105 sera and 51 samples of CSF. The control group included 21 sera that were donated from healthy adults and 20 samples of CSF from apparently healthy adults treated for traumatic fractures of the lower limbs, and the CSF control samples were collected from spinal anaesthesia. All samples were stored at − 80 °C until analysis.

Testing for anti-NMDAR antibody and anti-GABA_B_R antibody was performed by recombinant immunofluorescence using an IIFT: Glutamate Receptor Mosaic (Euroimmun, Lubeck, Germany). Paraneoplastic antibodies (i.e., anti-amphiphysin, -CV2, -PNMA2 (Ta), -Ri, -Yo, -Hu, -SOX1, -Titin, -Recoverin) were determined in an immunoblot assay using EUROLINE Neuronal Antigens Profile (IgG) (Euroimmun, Lubeck, Germany). CXCL10, CXCL13, IL10, and BAFF were analysed using a premixed multiplex system (USA R&D Systems, Inc.) human magnetic luminex assays. TGF-β1 was tested using ELISA kits (USA R&D Systems, Inc.) and a human TGF-β1 immunoassay。 The testing was performed according to the manufacturer’s instructions. As reported [10, 12], sera were diluted in a 1:5 concentration in the provided buffers, and the CSF was assayed without dilution.

### Statistical analysis

Demographic information and clinical features are expressed as numbers (N) and percentages (%). Levels of cytokines/chemokines are presented as the median (min, max). The Fisher’s exact or Wilcoxon rank sum tests were used to test for significant differences among the different patient groups as appropriate. Variables associated with outcomes or relapses (*p* < 0.1) were included in the following multiple logistic regression analyses. We used a stepwise approach for variable evaluation (backward selection based on likelihood ratio) for the identification of relevant independent variables to be used in the regression model, with a *p* value of less than 0.05 indicating statistical significance. To facilitate the comparison of effect sizes between cytokines, cytokine distributions were standardized to a mean of 0 and a standard deviation [SD] of 1. We performed all the statistical analyses with SAS, version 9.4 (SAS Institute Inc., Cary NC, USA).

## Results

### Patients and clinical features

A total of 110 patients who tested positive for anti-neuron antibodies were identified in this study. The patients had a median age of 47 years (range: 0.8–78 years), and 54 (49.1%) of the patients were women. Fifty-two patients (47.3%) had an acute onset, 45 (40.9%) had a chronic onset and 13 (11.8%) had a subacute onset. Thirty-three patients (30%) were identified as having MRI (FLAIR)/T2 abnormalities (excluding ischaemic lesions), 52 (47.3%) had EEG/EMG abnormalities, 41 (37.3%) had a leucocytosis in the CSF (white blood cells > 5× 10^6^/L) and 30 (27.3%) had elevated CSF protein levels (> 450 mg/L) (Table 1).

**Table 1:** Patient demographics and clinical features , antibodies, tumours in different groups

		Total		AE		PNS		No tumour		Tumours		Good outcomes		Poor outcomes		No relapse		Relapses	
Total		110	######	43	######	67	######	70	######	40	######	73	(66.36%)	37	(33.64%)	77	######	33	######
Age	<18 years	26	######	18	######	8	(7.27%)	24	######	2	(1.82%)	25	(22.73%)	1	(0.91%)	15	######	11	######
	≥18 years	84	######	25	######	59	######	46	######	38	######	48	(43.64%)	36	(32.73%)	62	######	22	######
Sex	Men	56	######	19	######	37	######	37	######	19	######	36	(32.73%)	20	(18.18%)	40	######	16	######
	Women	54	######	24	######	30	######	33	######	21	######	37	(33.64%)	17	(15.45%)	37	######	17	######
Types of onset	Acute	52	######	34	######	18	######	36	######	16	######	43	(39.09%)	9	(8.18%)	31	######	21	######
	Sub-acute	13	######	4	(3.64%)	9	(8.18%)	10	(9.09%)	3	(2.73%)	9	(8.18%)	4	(3.64%)	9	(8.18%)	4	(3.64%)
	Chronic	45	######	5	(4.55%)	40	######	24	######	21	######	21	(19.09%)	24	(21.82%)	37	######	8	(7.27%)
MRI Flair/T2 abnormality	No	66	######	29	######	37	######	41	######	25	######	42	(38.18%)	24	(21.82%)	52	######	14	######
	Yes	33	######	12	######	21	######	23	######	10	(9.09%)	23	(20.91%)	10	(9.09%)	19	######	14	######
EEG/EMG abnormality	No	7	(6.36%)	5	(4.55%)	2	(1.82%)	5	(4.55%)	2	(1.82%)	6	(5.45%)	1	(0.91%)	3	(2.73%)	4	(3.64%)
	Yes	52	######	22	######	30	######	36	######	16	######	34	(30.91%)	18	(16.36%)	40	######	12	######
CSF leukocytosis	No	42	######	15	######	27	######	28	######	14	######	33	(30.00%)	9	(8.18%)	28	######	14	######
	Yes	41	######	23	######	18	######	28	######	13	######	24	(21.82%)	17	(15.45%)	28	######	13	######
Elevated CSF PRO	No	53	######	30	######	23	######	38	######	15	######	42	(38.18%)	11	(10.00%)	29	######	24	######
	Yes	30	######	8	(7.27%)	22	######	18	######	12	######	15	(13.64%)	15	(13.64%)	27	######	3	(2.73%)
CSF abnormality	No	31	######	14	######	17	######	22	######	9	(8.18%)	25	(22.73%)	6	(5.45%)	18	######	13	######
	Yes	52	######	24	######	28	######	34	######	18	######	32	(29.09%)	20	(18.18%)	38	######	14	######
Anti-neuron antibodies	Anti-NMDAR	42	######	42	######	0	(0.00%)	35	######	7	(6.36%)	36	(32.73%)	6	(5.45%)	23	######	19	######
	Anti-Hu	19	######	0	(0.00%)	19	######	7	(6.36%)	12	######	6	(5.45%)	13	(11.82%)	14	######	2	(1.82%)
	Anti-Yo	14	######	0	(0.00%)	14	######	9	(8.18%)	5	(4.55%)	6	(5.45%)	8	(7.27%)	10	(9.09%)	4	(3.64%)
	Anti-PNMA2	12	######	0	(0.00%)	12	######	8	(7.27%)	4	(3.64%)	10	(9.09%)	2	(1.82%)	10	(9.09%)	2	(1.82%)
	Anti-CV2	8	(7.27%)	0	(0.00%)	8	(7.27%)	2	(1.82%)	6	(5.45%)	4	(3.64%)	4	(3.64%)	7	(6.36%)	1	(0.91%)
	Anti-Amphiphysin	8	(7.27%)	0	(0.00%)	8	(7.27%)	2	(1.82%)	6	(5.45%)	4	(3.64%)	4	(3.64%)	7	(6.36%)	1	(0.91%)
	Anti-SOX1	7	(6.36%)	0	(0.00%)	7	(6.36%)	4	(3.64%)	3	(2.73%)	3	(2.73%)	4	(3.64%)	5	(4.55%)	2	(1.82%)
	Others	7	(6.36%)	1	(0.91%)	6	(5.45%)	4	(3.64%)	3	(2.73%)	7	(6.36%)	0	(0.00%)	4	(3.64%)	3	(2.73%)
Tumours	Non-small cell lung cancer	9	(8.18%)	0	(0.00%)	9	(8.18%)	0	(0.00%)	9	(8.18%)	1	(0.91%)	8	(7.27%)	9	(8.18%)	0	(0.00%)
	Small cell lung cancer	6	(5.45%)	1	(0.91%)	5	(4.55%)	0	(0.00%)	6	(5.45%)	2	(1.82%)	4	(3.64%)	5	(4.55%)	1	(0.91%)
	Breast cancer	5	(4.55%)	0	(0.00%)	5	(4.55%)	0	(0.00%)	5	(4.55%)	1	(0.91%)	4	(3.64%)	3	(2.73%)	2	(1.82%)
	Ovarian teratoma	3	(2.73%)	3	(2.73%)	0	(0.00%)	0	(0.00%)	3	(2.73%)	3	(2.73%)	0	(0.00%)	1	(0.91%)	2	(1.82%)
	Others	18	######	3	(2.73%)	15	######	0	(0.00%)	15	######	9	(8.18%)	9	(8.18%)	13	######	5	(4.55%)

Data are n (%)

*AE* autoimmune encephalitis, *PNS* paraneoplastic neurological syndromes, *MRI FLAIR* Magnetic resonance imaging fluid-attenuated inversion recovery, *EEG* electroencephalogram, *EMG* electromyogram, *CSF* cerebrospinal fluid, *PRO* protein, *NMDAR* N-methyl-D-aspartate receptor

Regarding the anti-neuron antibodies in the patients, 42 patients (38.2%) tested positive for anti-NMDAR antibodies, 19(17.3%)for anti-Hu, 14 (12.7%)for anti-Yo, 12 (10.9%) for anti-PNMA2, 8(7.3%) for anti-Amphiphysin, 8 (7.3%) for anti-CV2 and 7 for others (6.4%). As reported [13], 43 (39.1%) patients (42 for anti-NMDAR and 1 for anti-GABA_B_R) were classified as AE, and the other 67 patients (60.9%) were classified as PNS. Factors significantly associated with PNS patients included having a chronic onset (*P* < 0.0001), an elevated CSF protein level (*P* = 0.0116), tumours (*P* = 0.0005), no relapse (*P* = 0.0113) and poor outcomes (*P* = 0.0004) when compared to AE patients. In this study, 40(36.4%) patients had a tumour. Nine tumours (8.2%) were non-small cell lung cancers, 6 (5.5%) were small cell lung cancers, 5 (4.5%) were breast cancers, 3 (2.7%) were ovarian teratomas and 17 (15.5%) were other tumours.

At the final follow-up, 37 patients (33.6%) had poor outcomes. In patients with the poor outcomes, we recorded significant relevant factors, including having a chronic onset (*P* = 0.0002), an elevated CSF protein level (*P* = 0.0075), tumours (*P* < 0.0001) and no relapse (*P* = 0.0081). During the follow-up, 33 patients (30%) had clinical relapses, including 23 patients (23/30, 76.7%) that relapsed once and 1 patient that relapsed 8 times. We also recorded significant positive correlations between having a relapse and an acute onset (*P* = 0.0163), MRI (FLAIR)/T2 abnormalities (*P* = 0.0345) and a normal CSF protein level (*P* = 0.0012).

### Analysis of cytokines/chemokines

**Fig. 1:**
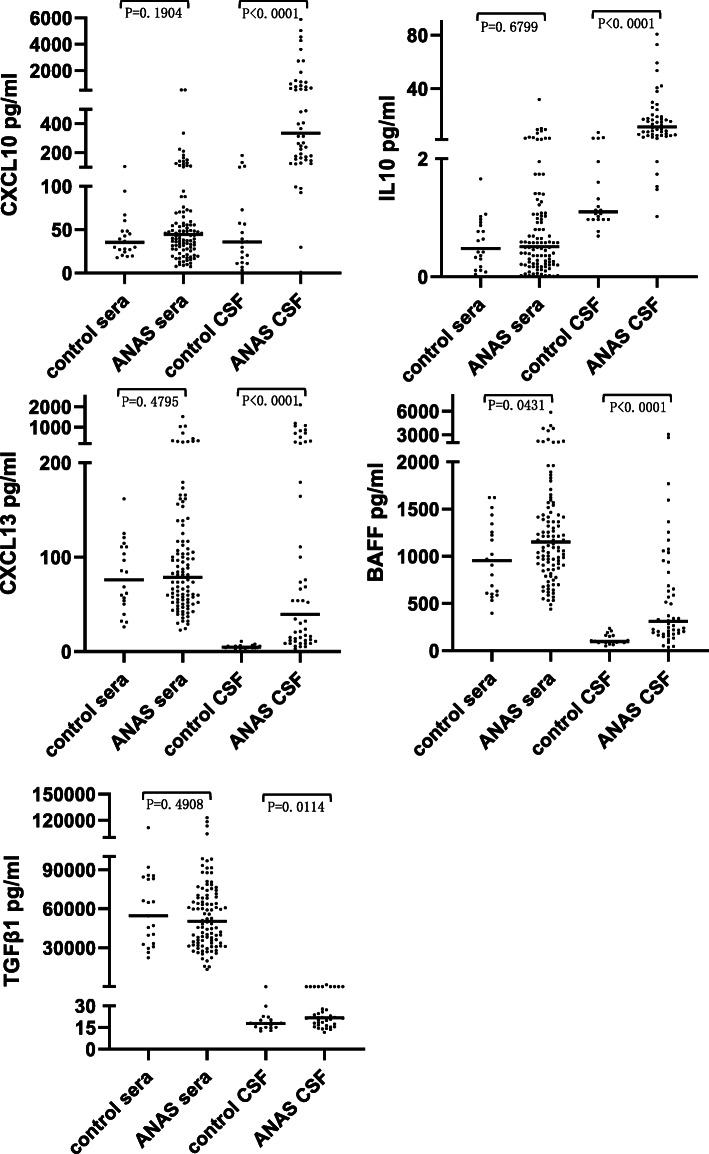
Cytokines/chemokines levels in sera and CSF compared between the ANAS and control group. There were significant differences in serum BAFF levels (*P* = 0.0431) and in CSF CXCL10, CXCL13, IL10, BAFF and TGFβ1 levels (*P* < 0.05) between the ANAS and control group

In this study, we collected 105 serum samples and 51 samples of CSF from ANAS patients and 21 serum samples and 20 samples of CSF from the control group. There were significant differences in serum BAFF levels (*P* = 0.0431) and in CSF CXCL10, CXCL13, IL10, BAFF and TGFβ1 levels (*P* < 0.05) between the ANAS and control group (Fig. 1).

**Fig. 2:**
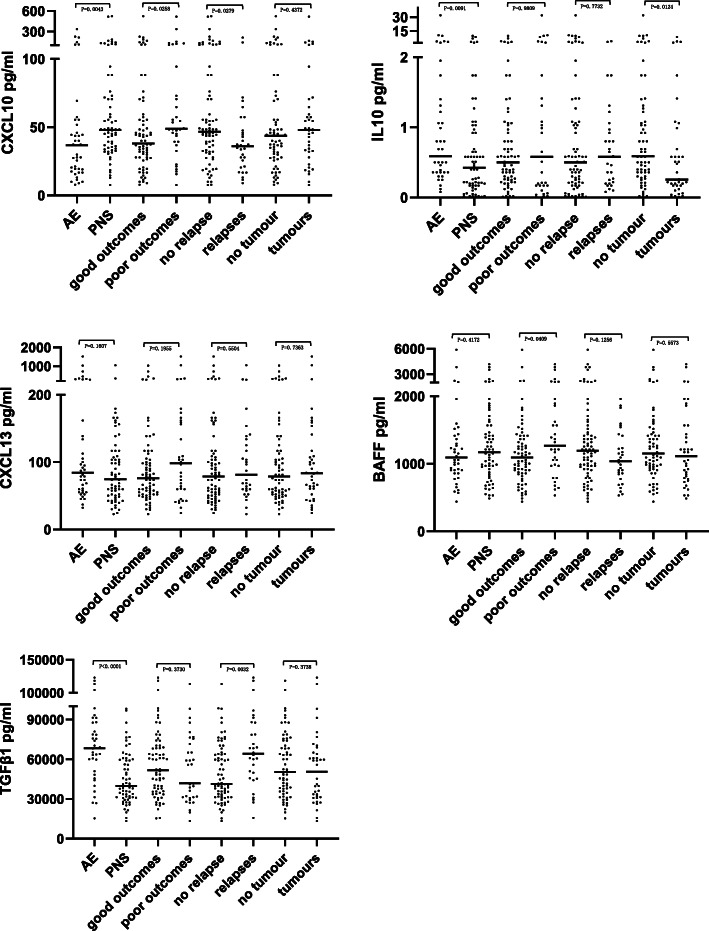
Cytokines/chemokines levels in sera compared within different ANAS groups. In AE compared to PNS, there were significant differences in CXCL10 (*P* = 0.0043), IL10 (*P* = 0.0091) and TGFβ1 (*P* < 0.0001). There were significant inverse relationships between IL10 levels and tumours (*P* = 0.0124). And significant positive correlations between CXCL10 (*P* = 0.0258), BAFF (*P* = 0.0409) levels and poor outcomes. And significant positive correlations for TGFβ1 levels (*P* = 0.0032), significant inverse relationships for CXCL10 levels (*P* = 0.0279) in patients with relapses

In the univariate analysis of the serum cytokines/chemokines levels, there were significant differences in CXCL10 (*P* = 0.0043), IL10 (*P* = 0.0091) and TGFβ1 (*P* < 0.0001) between the AE and PNS patients. We detected significant inverse relationships between IL10 levels and patients with tumours (*P* = 0.0124). In patients with poor outcomes, the significant relevant factors included higher levels of CXCL10 (*P* = 0.0258) and BAFF (*P* = 0.0409) than those with good outcomes. In patients with relapses, we recorded significant positive correlations for TGFβ1 (*P* = 0.0032), whereas we detected significant inverse relationships for CXCL10 (*P* = 0.0279) compared with no relapse (Fig. 2). For the CSF cytokines/chemokines levels, there were no significant differences between the different groups (*P* > 0.05).

### Predictors of outcomes and relapses

In all patients, the univariate analysis revealed factors associated with poor outcomes, including having a chronic onset (*P* = 0.0002), an elevated CSF protein level (*P* = 0.0075), tumours (*P* < 0.0001), no relapse (*P* = 0.0081), PNS (*P* = 0.0004), higher levels of serum CXCL10 (*P* = 0.0258) and serum BAFF (*P* = 0.0409) (Table 2). Then, the logistic multivariate stepwise regression analysis indicated the factors that were associated with poor outcomes, and these included having tumours (OR 10.156, CI 1.458–70.748, *P* = 0.0193) and a chronic onset (OR 13.921, CI 1.279–151.586, *P* = 0.0306) (Table 4). As mentioned above, in the univariate analysis, factors associated with relapses included having a chronic onset (*P* = 0.0163), MRI (FLAIR)/T2 abnormalities (*P* = 0.0345), a normal CSF protein level (*P* = 0.0012), AE (*P* = 0.0113), a good outcome (*P* = 0.0081), lower levels of serum CXCL10 (*P* = 0.0279) and higher levels of serum TGFβ1 (*P* = 0.0032) (Table 2). In the multivariate analysis, none of the factors was associated with relapses (*P* > 0.05).

**Table 2:** In 110 ANAS patients, factors associated with outcomes or relapses in univariate analysis

Outcomes				Relapses			
	Good outcomes	Poor outcomes	*p*		NO relapose	Relapses	*p*
Types of onset			0.0002	Types of onset			0.0163
Acute	43(58.90%)	9(24.32%)		Acute	31(40.26%)	21(63.64%)	
Sub-acute	9(12.33%)	4(10.81%)		Sub-acute	9(11.69%)	4(12.12%)	
Chronic	21(28.77%)	24(64.86%)		Chronic	37(48.05%)	8(24.24%)	
Elevated CSF PRO			0.0075	Elevated CSF PRO			0.0012
No	42(73.68%)	11(42.31%)		NO	29(51.79%)	24(88.89%)	
Yes	15(26.32%)	15(57.69%)		YES	27(48.21%)	3(11.11%)	
Tumours			<0.0001	MRI Flair/T2 abnormalities			0.0345
No	57(81.43%)	13(18.57%)		NO	52(73.24%)	14(50.00%)	
Yes	16(40.00%)	24(60.00%)		YES	19(26.76%)	14(50.00%)	
AE or PNS			0.0004	AE or PNS			0.0113
AE	37(50.68%)	6(16.22%)		AE	24(55.81%)	19(44.19%)	
PNS	36(49.32%)	31(83.78%)		PNS	53(79.10%)	14(20.90%)	
Serum CXCL10	38.02(7.84-525.63)	48.77(7.58-516.68)	0.0258	Serum CXCL10	46.73(7.58-525.63)	36.11(9.00-211.62)	0.0279
Serum BAFF	1094(440.36-5882.00)	1266.5(531.88-4173.00)	0.0409	Serum TGFβ1	41401.57(13345.38-113337.9)	64140.37(15755.87-122994.8)	0.0032
CSF leukocytosis			0.0606				
No	33(57.89%)	9(34.62%)					
Yes	24(42.11%)	17(65.38%)					
CSF abnormality			0.0889				
No	25(43.86%)	6(23.08%)					
Yes	32(56.14%)	20(76.92%)					

Counting data are n (%), and measruement data are median (min, max)

*CSF* cerebrospinal fluid, *PRO* protein, *AE* autoimmune encephalitis, *PNS* paraneoplastic neurological syndromes, *CXCL* C-X-C motif chemokine, *BAFF* B cell activating factor, *MRI FLAIR* Magnetic resonance imaging fluid-attenuated inversion recovery, *TGFβ1* transforming growth factor β1, *ANAS* anti-neuron antibody syndrome

In the 51 patients with CSF cytokines/chemokines data, the ratios of two cytokine/chemokine levels were possible factors that might be associated with outcomes or relapses. In the univariate analysis, factors associated with poor outcomes included having higher levels of CSF BAFF (*P* = 0.0606), a chronic onset (*P* = 0.0010), an elevated CSF PRO level (*P* = 0.0123), tumours (*P* < 0.0001), and PNS (*P* = 0.0372) (Table 3). In the multivariate analysis, the factor associated with poor outcomes was tumours (OR 13.859, CI 2.242–85.683, *P* = 0.0047) (Table 4). In the univariate analysis, factors associated with relapses included larger ratios of serum TGFβ1/CSF CXCL10 (*P* = 0.0048), serum TGFβ1/serum CXCL13 (*P* = 0.0455), serum TGFβ1/serum BAFF (*P* = 0.0279), CSF TGFβ1/serum TGFβ1 (*P* = 0.0205), serum TGFβ1/CSF BAFF (*P* = 0.0076), higher levels of serum TGFβ1 (*P* = 0.0202) and other factors were identified that might be associated with relapses, including having a normal CSF protein level (*P* = 0.0632), AE (*P* = 0.0737), lower levels of CSF IL10 (*P* = 0.0913) and CSF BAFF (*P* = 0.0990) (Table 3). In multivariate analysis, the factors associated with relapses included lower levels of CSF BAFF (OR 0.038, CI 0.001–0.988, *P* = 0.0491) and a larger ratio of serum TGFβ1/serum CXCL13 (OR 5.809, CI 1.349–25.002, *P* = 0.0182) (Table 4).

**Table 3:** In 51 ANAS patients with CSF, factors associated with outcomes or relapses in univariate analysis

Outcomes				Relapses			
	Good outcomes	Poor outcomes	*p*		No relapse	Relapses	*p*
Elevated CSF PRO			0.0123	Elevated CSF PRO			0.0632
No	29(76.32)	4(33.33)		No	17(54.84)	16(84.21)	
Yes	9(23.68)	8(66.67)		Yes	14(45.16)	3(15.79)	
AE or PNS			0.0372	AE or PNS			0.0737
AE	28(71.79)	4(33.33)		AE	16(51.61)	16(80.00)	
PNS	11(28.21)	8(66.67)		PNS	15(48.39)	4(20.00)	
Tumours			<0.0001	CSF IL10	14.99(1.48-80.63)	7.8(1.02-28.29)	0.0913
No	36(92.31)	4(33.33)					
Yes	3(7.69)	8(66.67)					
Types of onset			0.001	Serum TGFβ1	52379.98(15370.86-113337.9)	69013.08(27451.21-118364.7)	0.0202
Acute	32(82.05)	4(33.33)					
Sub-acute	2(5.13)	1(8.33)					
Chronic	5(12.82)	7(58.33)					
CSF BAFF	267.68(2.54-3064.00)	742.31(89.23-2670.00)	0.0606	CSF BAFF	370.9(2.54-3064.00)	242.77(36.56-1367.00)	0.099
				Serum TGFβ1/CSF CXCL10	102.48(7.33-927.55)	310.13(6.02-3235.45)	0.0048
				Serum TGFβ1/serum CXCL13	574.07(70.48,2913.36)	1041.71(264.89,1618.54)	0.0455
				Serum TGFβ1/serum BAFF	41.76(5.34-113.62)	64.24(21.85-140.59)	0.0279
				CSF TGFβ1/serum TGFβ1	0.00064(0.00012-0.049)	0.00025(0.00014-0.0016)	0.0205
				Serum TGFβ1/CSF BAFF	114.29(16.12-1043.37)	359.88(55.28-2646.06)	0.0076

Counting data are n (%), and measruement data are median (min, max)

*CSF* cerebrospinal fluid, *PRO* protein, *AE* autoimmune encephalitis, *PNS* paraneoplastic neurological syndromes, *BAFF* B cell activating factor, *IL* interleukin, *TGFβ1* transforming growth factor β1, *CXCL* C-X-C motif chemokine, *ANAS* anti-neuron antibody syndrome

**Table 4:** Predictors of outcomes or relapses in ANAS patients in multivariate analysis

In 110 ANAS patients			In 51 ANAS patients with CSF		
Outcomes	OR(95%CI)	*p*	Outcomes	OR(95%CI)	*p*
Tumours	10.156(1.458-70.748)	0.0193	Tumours	13.859 (2.242-85.683)	0.0047
Types of onset	13.921(1.279-151.586)	0.0306			
			Relapses	Per-SD increase [OR(95%CI)]	*p*
			serum TGFβ1/serum CXCL13	5.809 (1.349-25.002)	0.0182
			CSF BAFF	0.038(0.001-0.988)	0.0491

*ANAS* anti-neuron antibody syndrome, *OR* odds ratio, *CI* confidence interval, *CSF* cerebrospinal fluid, *SD* standard deviation, *TGFβ1* transforming growth factor β1, *CXCL* C-X-C motif chemokine, *BAFF* B cell activating factor

## Discussion

Neuroimmunology is a rapidly expanding field in neuroscience that has led to a paradigmatic shift in the understanding of immunological diseases in the CNS and has led to the discovery of a series of new antibodies and neurological syndromes. This study was a retrospective analysis based on the clinical data of our centre in the past 7 years, which included some new insights to anti-neuron antibodies and related neurological syndrome. ANAS is a clinical neurological syndrome that is characterized by the presence of antibodies that target neuronal epitopes. As reported, the target antigens include nuclear or cytoplasmic proteins, such as Hu, Ri, Yo, Ma2, ANNA-3, MAP1B, SOX1, CRMP5, ZIC4, NIF, ITPR1 [4, 14], intracellular synaptic proteins, such as 65 kDa glutamic acid decarboxylase (GAD65) and amphiphysin, and cell-surface or synaptic proteins, such as NMDAR, AMPAR, γ-aminobutyric acid receptor-B (GABAR), LGI1, Caspr2, GIyR, mGIuR5 [4], DPPX [1], DR2 [2], Iglon5 [3] and neurexin-3α [15]. The main syndromes are classified as AE and PNS, which is based on the widely recognized methods in recent years [14, 16, 17]. The term ANAS is not intended to be used as a clinical diagnosis, but the recognition of this syndrome is useful for the understanding of the common aetiologies and pathogeneses. This study aimed to summarize the general characteristics and clarify the clinical range of ANAS, which is beneficial for clinical neuroscientists to understand the aetiological relationships and pathogeneses of diseases associated with ANAS.

The study clarified the clinical range of ANAS, summarized the data, including clinical features, outcomes and relapses, and then focused on the cytokines/chemokines found in the sera and CSF, so as to provide the basis for doctors’ clinical decision-making and to help analyse the related factors of the prognosis. In the univariate and logistic multivariate stepwise regression analyses, the study revealed that possible factors that were associated with poor outcomes included tumours and having a chronic onset, and possible factors associated with relapses included having lower levels of CSF BAFF and a larger ratio of serum TGFβ1/serum CXCL13. These factors might be predictors for ANAS.

In these cases, 47.3% of patients had acute onset, 30% had MRI FLAIR/T2 abnormalities, 47.3% had EEG/EMG abnormalities and 27.3% had elevated CSF protein. In a cohort study of anti-NMDAR encephalitis [18], MRI of the brain and EEG and CSF studies were abnormal in 33%, 90% and 79% of patients, respectively. The results suggested that these clinical features might not be specific for ANAS, and the diagnosis might require antibody detection.

There were more patients (60.9%) classified as PNS, and the common antibodies found were anti-NMDAR, anti-Hu and anti-Yo. A total of 36.4% of patients had tumours, and the majority of the tumours were lung cancers and breast cancers. As shown in other research on PNS, the common antibodies and tumours were anti-Yo, anti-Hu, and anti-titin antibodies and thymoma and lung cancers, respectivel y[19], which were partially consistent with our study. The inconsistency may have something to do with the different populations studied, including differences in the participants’ races. There were 66.4% of patients with good outcomes and 70% without relapses in our study, which indicated a relatively good prognosis for ANAS.

In general, ANAS is considered to be an immune-mediated disorder and has certain antibodies targeting neuronal epitopes. T and B cells might be responsible for the syndrome and participate in key steps [4]. Some cytokines/chemokines might play significant roles in the occurrence and development of ANAS by affecting the functions of T and B cells. Among anti-inflammatory factors, TGF-β1 and IL-10 play important roles in the development of inflammation in the peripheral and central nervous systems. TGF-β1 exerts its suppressive effects by inhibiting cytotoxic T cells, Th1 and Th2 cell differentiation and controlling B cell proliferation, survival, activation and differentiation [7]. IL-10 plays a pivotal role in restraining the immune response on myeloid cells by suppressing proinflammatory cytokines and antigen-presenting cells (APCs) [8]. In proinflammatory elements, CXCL13, CXCL10 and BAFF play crucial roles in the survival, activation and recruitment of T or B cells. CXCL13 is considered to be the major determinant for B cell recruitment to the CNS compartment in neuroinflammatory diseases, especially in the CSF [9]. It is also mildly elevated in multiple sclerosis (MS) and neuromyelitis optica [20, 21]. CXCL10 recruits CXCR3^+^ cells, such as activated T cells, in CNS inflammation,[10] and they were found to colocalize with active MS lesions [22]. BAFF is a member of the tumour necrosis factor (TNF) family and acts as an amplifier of autoimmune responses by promoting the activation and survival of B cells [11]. The accumulated data suggest that the above factors might interact with T and B cells and affect the pathogenesis of ANAS

The measurement of cytokines/chemokines in the sera and CSF were the key points of the study. Our study aimed to screen several cytokines/chemokines as predictors of the outcomes and relapses of ANAS. As reported in previous studies [10, 23], PNS patients had high levels of the chemokine CXCL10 in the CSF. Elevated soluble Fas and FasL have been found in the CSF and sera of patients with anti-NMDAR encephalitis [13]. Certain key cytokines/chemokines might act as biomarkers of inflammation and the response to treatments in the CNS [12, 24]. These studies demonstrated that some cytokines/chemokines may be related to ANAS and influence the prognosis of this syndrome.

In this study, some cytokines/chemokines related to the activation or inhibition of T and B cells were observed. We tested the levels of cytokines/chemokines in the CSF and sera and then further investigated the relationship between cytokines/chemokines and ANAS. In the univariate analysis, the elevation of CXCL10 and BAFF in the sera indicated a poor outcome, and a decrease of CXCL10 and an elevation of TGFβ1 in the sera was associated with relapses. In the multivariate analysis, the factors associated with relapses included lower levels of CSF BAFF and a larger ratio of serum TGFβ1/serum CXCL13. These cytokines/chemokines might be predictors for outcomes and relapses of ANAS. In this research, we aimed to identify some cytokines/chemokines that were able to predict the prognosis. We also explored more associated factors and calculations that might be related to the prognosis. The ratios of two cytokine/chemokine levels were counted as factors. Then, we observed whether they were associated with outcomes and relapses in the univariate and multivariate analyses. In our study, the ratio of serum TGFβ1/serum CXCL13 was associated with relapses. Multicentre clinical trials and verifications with larger samples might be helpful in order to clarify the relationship between these ratios and prognosis of ANAS. These ratios in this study may indicate a novel way of determining prognosis. In this study, we mainly observed the phenomenon including the difference in factor level in different ANAS groups and between ANAS and normal population. In the future experiment, we will analyse the function and quantity of T and B cell subsets by flow cytometry. At the same time, the interaction in different inflammatory factors would be studied to explore the mechanism in depth.

There were many factors associated with the outcomes and relapses of many diseases, and some clinical features have been reported in previous studies. Conscious disturbances and CSF antibody titres have been shown to be predictors of poor outcomes in anti-NMDAR encephaliti s[25], and aggressive immune therapy might reduce the risk of relapses [26]. In our study, the clinical data and cytokines/chemokines in the sera in all ANAS patients were screened as possible predictors during the univariate and multivariate analyses. The factors associated with poor outcomes included having tumours and a chronic onset. No factor was associated with relapses. Meanwhile, we selected all 51 ANAS cases with CSF data and analysed the clinical data and cytokines/chemokines in the sera and CSF. Having tumours was also associated with poor outcomes. The factors associated with relapses included CSF BAFF and the ratio of serum TGFβ1/serum CXCL13. These factors might be linked to the prognosis of ANAS. Thus, to evaluate prognosis of the disease more comprehensively and objectively, we analysed the associated factors including clinical features and cytokines/chemokines.

Much evidence has been collected in previous studies of AE and PNS [14, 17], and the pathogenesis of neuronal autoantigens has been explored [4]. With the development of neuroimmunology, more antibodies to neuronal autoantigens might be discovered, which may lead to a deeper understanding into the immune mechanisms involved in AE and PNS. In this study, we proposed the concept of “anti-neuronal antibody syndrome” to clarify the pathogenetic relationships. The clinical and immunological aspects of ANAS have been clarified, which might contribute to neuroscientists conducting more profound studies regarding clinical syndromes. Here, we report the data of clinical features and cytokines/chemokines in the sera and CSF, and the data regarding the antibody titres and clinical treatments will be discussed in future studies. Our study focused on the disease on a class of antibodies that target common neuronal epitopes, which was distinguished from most current researches emphasizing one specific antibody. This might promote the further study of ANAS and provide a new idea on clinical study in neuroimmunology.

Our study had the limitations of not being randomized, having fewer cases and having a lack of complete data regarding the cytokines/chemokines in the CSF, which reduces the level of evidence in this ANAS study. Some other factors might influence the levels of cytokines/chemokines found in the sera and CSF, including sampling time, storage conditions, test method, etc. The interactions with different cytokines/chemokines are complex pathological processes. In particular, there is a lack of data on the function and quantity of T and B cells. It is difficult to reflect the complex changes in the process of disease solely using the levels or ratios of cytokines/chemokines in the sera or CSF. However, we proposed the clinical range of ANAS, summarized the features and cytokines/chemokines in the sera and CSF, and then screened possible predictors associated with outcomes and relapses. This might be helpful for the in-depth study of ANAS.

Our study focused on studying the association of disorders with a class of antibodies targeting common neuronal epitopes, and this study highlights the fact that ANAS is a clinical neurological syndrome, not a clinical diagnosis. The term ANAS was proposed for the first time and the clinical range was clarified. The study of ANAS might be a beginning for the understanding of these conditions and should be further promoted. This might provide a novel idea for clinical study in neuroimmunology.

## Conclusion

Our study proposed the term ANAS and clarified the clinical range of this condition. We analysed the association between these diseases, the clinical features and levels of cytokines/chemokines. Then, we screened possible factors associated with poor outcomes, including having tumours, a chronic onset and factors associated with relapses, which included having lower levels of CSF BAFF and a larger ratio of serum TGFβ1/serum CXCL13. These factors might be related to the prognoses of the diseases.

## Data Availability

The datasets generated and analysed during the current study are available from the authors on reasonable request and provided a Data Sharing Agreement approved by the Danish Data Protection Agency.

## Abbreviations

ANAS: Anti-neuron antibody syndrome
AE: Autoimmune encephalitis
PNS: Paraneoplastic neurological syndrome
NMDAR: N-methyl-D-aspartate receptor
BAFF: B cell-activating factor
CSF: Cerebrospinal fluid
CXCL: C-X-C motif chemokine
TGFβ1: Transforming growth factor β1
EEG: Electroencephalogram
EMG: Electromyogram
mRS: Modified Rankin scale
MRI: Magnetic resonance imaging
FLAIR: Fluid-attenuated inversion recovery
GAD65: 65 kDa glutamic acid decarboxylase
GABAR: γ-aminobutyric acid receptor
